# Role of the Radiotherapy Boost on Local Control in Ductal Carcinoma *In Situ*

**DOI:** 10.1155/2012/748196

**Published:** 2012-04-08

**Authors:** Olivier Riou, Claire Lemanski, Vanessa Guillaumon, Olivier Lauche, Pascal Fenoglietto, Jean-Bernard Dubois, David Azria

**Affiliations:** ^1^Département d'Oncologie Radiothérapie, CRLC Val d'Aurelle-Paul Lamarque, 34298 Montpellier, France; ^2^Département de Recherche Clinique, CRLC Val d'Aurelle-Paul Lamarque, 34298 Montpellier, France

## Abstract

Ductal carcinoma *in situ* of the breast is associated with low mortality rates, but local relapse is a matter of concern in this disease. Risk factors for local relapse include young age, close or positive margins, and tumor necrosis. Whole breast irradiation following breast-conserving surgery for ductal carcinoma *in situ* significantly reduces the risk of local relapse as compared to breast-conserving surgery alone. Studies point to similar outcomes between breast-conserving surgery plus radiotherapy and mastectomy, in the absence of extensive disease. A complementary boost to the surgical bed improves outcomes for patients with invasive breast cancer. However, the effect of this strategy has never been prospectively reported for ductal carcinoma *in situ*. Two randomized controlled trials assessing this issue are ongoing. This paper represents an update on available literature about radiotherapy for DCIS with a special focus on the role of a radiotherapy boost to the tumor bed.

## 1. Introduction

Ductal carcinoma *in situ* (DCIS) is a proliferation of malignant cells inside galactophoric ducts without basal membrane invasion. Its incidence has dramatically increased in the recent years due to the widespread use of mammographic screening. Accounting for approximately 20 to 30% of the breast cancer cases [1], DCIS is heterogeneous in clinical presentation, varying from a palpable mass, mammographically detected tumor, or nipple discharge [2]. Despite a high cure rate, invasive recurrence and death may occur in case of insufficient local treatment. Patients with clinically large, multicentric, and extensive tumors are more likely to undergo mastectomy than breast-conserving surgery (BCS), because of a higher risk of recurrence [3]. Likewise, mastectomy may be the preferred strategy in case of diffuse suspicious-appearing microcalcifications in the breast, inability to obtain margin control by lumpectomy and/or reexcision(s), medical contraindication to irradiation, and when an unfavorable tumor-to-breast size ratio does not permit margin-negative lumpectomy with cosmetically acceptable results [4].

No randomized controlled trials comparing mastectomy with more conserving management are available, but different studies point to similar outcomes between BCS plus radiotherapy and mastectomy, whereas BCS alone tends to be inferior [5]. Therefore, BCS plus radiotherapy is an accepted strategy when mastectomy can be avoided, in view of the morbidity of radical surgery and the favorable prognosis of such patients. A number of randomized controlled trials of adjuvant radiotherapy have indeed demonstrated a reduced risk of both invasive and local recurrences, as well as a low risk of side effects [6, 7].

A radiation boost to the tumor bed has been shown to significantly improve local control in patients with invasive breast cancer [8, 9]. However, the usefulness of a boost has not been so well assessed in the setting of DCIS, and prospective studies are missing.

This paper aims at updating available literature on the subject and at developing the rationale for randomized multicenter phase 3 studies assessing the role of surgical bed boost following whole breast irradiation (WBI).

## 2. Risk Factors for Local Relapse following Breast-Conserving Therapy

Several studies have attempted to identify the local recurrence risk factors following breast-conserving surgery [10]. Clinical factors have been determined by uni- and multivariate analysis of randomized trials and multicenter retrospective studies. These findings suggest that a family history of breast cancer, a young-onset disease, and a palpable tumor of more than 1 cm are factors that may adversely affect local control [11–13]. Moreover, the comedocarcinoma subtype, a histopathology size greater than 10 mm, necrosis, and positive margins have been shown to be statistically significant predictive factors for recurrence in women under 40 years old [14].

Close or positive margins are considered to be risk factors for recurrence, whether patients are irradiated or not. In the B-24 and EORTC 10853 trials, although patients were supposed to have free margins for inclusion [15, 16], a central pathology review of the specimens found a significant amount of positive and unknown margins [17]. In these trials, positive margins were found to be independent factors for the development of local relapse after BCS [18]. According to the meta-analysis of Dunne et al., a margin threshold of 2 mm may be sufficient when BCS is combined with RT [19].

Apart from margins status, tumor necrosis appears to be an important relapse risk factor, even in multifactorial analysis [20]. The histological size, the Scarff-Bloom-Richardson grade, and the degree of differentiation are minor prognostic factors. No prognostic biological or genetic factors have been individualized so far in ductal carcinoma *in situ *[21].

## 3. The Influence of Adjuvant Radiotherapy in the Conserving Treatment of DCIS

The role of WBI is to reduce the rate of local relapse and to allow for breast conservation. Monocenter and multicenter retrospective studies with 5 to 15 years of followup found recurrence rates of about 7 to 17%. About half of recurrences were invasive, and the metastatic rate was less than 10%. Nevertheless, these studies had heterogeneous inclusion criteria, surgical treatment, radiation dose, and followup [22].

Four randomized controlled trials have assessed the role of irradiation in this setting. A worldwide collaboration group, the Early Breast Cancer Trialists' Collaborative Group (EBCTCG), has centrally reviewed these four trials to give an updated overview of the impact of radiotherapy in DCIS. The latest report confirmed a 15.2% reduction in the absolute 10-year risk of ipsilateral breast event, regardless of age at diagnosis, extent of breast-conserving therapy, use of tamoxifen, margin status, comedonecrosis, focality, grade, or tumor size. The amount of risk reduction was higher in older women (>50 years). However, no significant effect on overall mortality or breast cancer mortality was found after a followup of 10 years [7].

### 3.1. NSABP B17

The NSABP B17 trial was undertaken in the United States and Canada from 1985 to 1990. The results of this trial were first published in 1993 and then updated in 2001 [23, 24]. A total of 818 patients were randomized, after initial lumpectomy for DCIS, to either surveillance or WBI to 50 Gy in 25 fractions without tumor bed boost. After a mean followup of 129 months, a 57% reduction in the risk of invasive and *in situ* breast recurrences was seen. No differences in overall survival, metastatic, or contralateral cancer rates were shown. A pooled analysis of this study and of the NSABP B24 trial confirmed a 52% reduction in ipsilateral breast recurrence risk at 15 years when patients were offered radiation therapy [25].

### 3.2. EORTC 10853

This trial was conducted in Europe from 1986 to 1996 and accrued one thousand and ten DCIS patients with free margins after lumpectomy. First analysis after a followup of 4.2 years showed a significant reduction in invasive relapse risk, an increased risk of contralateral cancer, and equivalent survival and metastatic rates [17]. An update after a followup of 10.5 years confirmed these results, with a 48% reduction in intraductal recurrences and a 42% decrease in invasive relapses [18].

### 3.3. UK/ANZ DCIS Trial

This English study was a multicenter randomized trial which assessed both the influence of WBI and the role of tamoxifen adjuvant treatment following BCS. Randomization was independent for radiotherapy and tamoxifen, stratified by screening center, and blocked in groups of four [26]. One thousand seven-hundred and one patients were accrued and one thousand and thirty patients were randomized to radiotherapy or observation. This trial has been recently updated after a followup of 12.7 years [27]. The overall reduction in local relapse was 59%, and the ipsilateral invasive disease risk reduction was 68%. No difference in the number of contralateral cancer cases was seen. The risk reduction was similar whether patients had tamoxifen or not.

### 3.4. SweDCIS Trial

This multicenter Swedish trial enrolled DCIS patients treated by BCS with tumor-free margins. One thousand and forty-six women were randomized to either radiotherapy or no radiotherapy. Once again, the analysis after a followup of 5.2 years showed a 3-fold reduction in invasive and *in situ* local relapses [28]. An updated analysis published in 2008 confirmed a relative risk reduction of 60% in local recurrence [29]. Women more than 50 years seemed to benefit most from adjuvant radiotherapy in this trial, and the authors concluded that older age should not preclude DCIS women from radiotherapy.

## 4. The Role of the Radiotherapy Boost following BCS and WBI in DCIS

The rationale for dose escalation to the tumor bed relies on the frequent presence of residual tumor cells in a 10 mm radius of the tumor. A dose of 50 Gy does not seem to be high enough to kill such remaining cells. However, wider excisions with extensive margins significantly alter the cosmetic results. Margins greater than 2 or 3 mm are considered safe, despite a risk of remaining tumor cells of up to 20% [19, 22]. This risk legitimates dose escalation studies in DCIS.

Several retrospective studies have tried to assess the role of a boost following WBI for DCIS.

An international multicenter retrospective study was performed on 373 patients, aged 45 years or less, treated in 18 institutions [30]. Forty five percent of them underwent WBI, 40% underwent WBI plus a boost, and 15% had no radiotherapy. The relapse-free survival rates at ten years were 46% without radiotherapy, 72% in the WBI group, and 86% in the WBI plus boost group. Differences were statistically significant with an overall risk reduction for local relapse of 66% with WBI and 85% with WBI and boost. In multivariate analysis, the margins status and the radiation dose were the only two independent factors for relapse-free survival. No difference in overall survival was found.

Another study by Wong et al. confirmed the favorable effect of the radiation boost, with no local relapse observed among 79 patients receiving a boost, whereas 8 of 141 patients in the “no-boost” group experienced in-breast local recurrence [31]. These results were obtained despite a higher risk for local relapse in the boost group: 48% (boost group) *versus* 8% (no boost group) had positive or less than 1 mm margins.

On the contrary, other reports found no difference in local relapse according to the total radiation dose level: <60 Gy *versus *>60 Gy [32], <60 Gy *versus *60*–*66 Gy* versus *>66 Gy [13, 33, 34]. In the study by Wai et al., only 50% of patients had radiotherapy after BCS, including 35% receiving WBI without boost and 15% receiving WBI plus a boost to the lumpectomy bed [35]. Moreover, partial breast boost was used primarily in subjects with positive or close margins, which could alter the outcome of these patients and explain these different results. If a retrospective cohort study of 208 DCIS patients with close or focally involved margins after BCS appeared to show a similar effect of a 16-Gy boost to that of a reexcision [36], prospective studies are warranted to confirm this finding before reexcision may be avoided in all cases.

## 5. Ongoing Clinical Trials and Future Directions

Two randomized controlled trials addressing the role of a 16 Gy boost are ongoing, and patients are currently being recruited.

An international trial of the Trans-Tasman Radiation Oncology Group, started in 2008, simultaneously evaluates the role of the boost to the tumor bed and the effect of hypofractionation on outcomes in DCIS. Patients are divided into 3 strata and randomized within those groups: group A is designed to evaluate the effect of the boost and of the fractionation schedule, group B, the boost after 50 Gy in 25 fractions of WBI, and group C assesses the interest of the boost after 42.5 Gy in 16 fractions and 22 days of WBI.

In the French multicenter prospective randomized Bonbis trial, DCIS women aged 18 and over are randomly assigned to a 16 Gy boost or no boost, following BCS and the delivery of 50 Gy in 25 fractions to the whole breast [37]. Nearly half of the patients have been enrolled so far (900 of the planned 1950 women). Inclusion criteria include tumor-free margins, the presence of surgical clips in the lumpectomy bed to ease the boost delivery, and no past history of cancer. A centralized review of all specimens is performed, as well as an assessment of dummy runs and technical radiotherapy cases. Radiotherapy should start within 12 weeks of surgery. Translational studies investigating candidate genes and a predictive test of late toxicity will hopefully help to individualize patients who can be safely treated with dose escalation to the tumor bed.

## 6. Summary

BCS plus WBI is an accepted strategy for DCIS when mastectomy can be avoided. The effect of a complementary boost to the tumor bed has never been prospectively assessed. Two ongoing randomized controlled trials addressing this issue should help to individualize patients who may benefit from this treatment.
